# Arterial stiffness correlates with progressive nailfold capillary microscopic changes in systemic sclerosis: results from a cross-sectional study

**DOI:** 10.1186/s13075-019-2051-3

**Published:** 2019-11-27

**Authors:** Stergios Soulaidopoulos, Eleni Pagkopoulou, Niki Katsiki, Eva Triantafyllidou, Asterios Karagiannis, Alexandros Garyfallos, George D. Kitas, Theodoros Dimitroulas

**Affiliations:** 1 Fourth Department of Internal Medicine, Hippokration General Hospital, Medical School, Aristotle University of Thessaloniki, Konstantinoupoleos Str. 49, Thessaloniki, Greece; 2 Second Propedeutic Department of Internal Medicine, Hippokration General Hospital, Medical School, Aristotle University of Thessaloniki, Thessaloniki, Greece; 3 Arthritis Research UK Centre for Epidemiology, University of Manchester, Manchester, UK; 4 Department of Rheumatology, Dudley Group NHS Foundation Trust, Dudley, UK

**Keywords:** Capillaroscopy, Systemic sclerosis, Microangiopathy, Arterial stiffness, Arteriosclerosis

## Abstract

**Background:**

While microangiopathy is well-documented in systemic sclerosis (SSc), a potential link between SSc and macrovascular disease is highly debated and remains to be established. The aim of the present study is to investigate the association between micro- and macrovascular involvement in the setting of SSc.

**Methods:**

Consecutive, consenting SSc patients were assessed by nailfold video-capillaroscopy (NVC) to evaluate the microcirculation. The number of capillaries per mm^2^ and the capillaroscopic skin ulcer risk index (CSURI) were measured, and findings were also classified into three scleroderma patterns (i.e., early, active, and late). Carotid intima-media thickness (IMT), aortic augmentation index corrected for a heart rate of 75 beats per minute (AIx-75), carotid-femoral pulse wave velocity (PWV), and central systolic and diastolic blood pressure were also determined to assess macrovascular function.

**Results:**

A total of 37 patients were studied. A significant correlation was observed between AIx and the average number of capillaries per mm^2^ (*r* = − 0.34, *p* = 0.047) and between AIx and CSURI (*r* = 0.35, *p* = 0.044). Patients with the “early” scleroderma pattern had lower AIx values compared with “active” (20.5 ± 11.4 vs 34.1 ± 11.5%, *p* = 0.02) and “late” (20.5 ± 11.4 vs 33.4 ± 8.8%, *p* = 0.05) patterns. No other significant correlations were found between macrovascular biomarkers (PWV, carotid IMT, systolic and diastolic central blood pressure) and the capillaroscopic measurements.

**Conclusions:**

These data suggest that arterial stiffness (as assessed by AIx-75) correlates with microvascular damage in patients with SSc.

## Introduction

Systemic sclerosis (SSc) is a heterogeneous connective tissue disorder characterized by extensive fibrosis of the skin and progressive multi-organ involvement [1]. Its pathogenesis is complex encompassing interrelations between inflammation and autoimmune activation, connective tissue remodeling, dysregulation, and vascular dysfunction [2]. From a pathophysiological standpoint, microvascular damage represents the earliest morphological and functional process of the disease which is present in early asymptomatic stages or may manifest clinically as Raynaud’s phenomenon several years before the diagnosis [3].

Nailfold video-capillaroscopy (NVC) is an established, non-invasive and reproducible method for the assessment of microcirculation [4]. It represents a reliable tool for the detection and classification of typical microvascular changes which are generally described under the term “scleroderma pattern” ranging from enlarged and giant capillaries with the presence of edema and capillary hemorrhages, to extensive capillary loss and complete disorganization of the normal capillary distribution [5]. In addition to the established diagnostic value of NVC [6], associations between capillaroscopic alterations and cardiopulmonary complications of SSc suggest an intriguing role of NVC as a potential prognostic tool for the presence and severity of internal organ involvement [7].

While microangiopathy is well-documented in SSc, a potential link between SSc and macrovascular disease is highly debated and is yet to be established. Results from observational retrospective studies have revealed a high relative risk for cardiovascular (CV) events among SSc patients [8, 9]. However, the relationship between macrovascular atherosclerotic markers namely carotid intima-media thickness (cIMT), pulse wave velocity (PWV) and NVC patterns has not been adequately studied in SSc individuals. Only few studies have addressed this question, providing limited data regarding the association between cIMT [10], brachial artery flow-mediated dilatation (FMD) [11], arterial stiffness [12], and NVC abnormalities. These studies suggest a link between micro- and macrovascular alterations in SSc.

The aim of the present study was to explore whether typical SSc-related NVC abnormalities and patterns are associated with indices of macrovascular disease in patients with SSc.

## Materials and methods

### Patients

SSc patients attending the Scleroderma Clinic of the Fourth Department of Internal Medicine, Hippokration General Hospital, Thessaloniki, Greece, between September 2016 and June 2017 were recruited. All participants fulfilled the revised EULAR/ACR criteria for the diagnosis of SSc [6]. Individuals with documented CV disease defined as history of myocardial infarction, percutaneous transluminal coronary angioplasty or coronary artery bypass graft surgery, stroke or transient ischemic attack or carotid artery surgical procedures as well as patients with history of chronic kidney disease, diabetes mellitus, peripheral artery disease, and active smokers were excluded. The study was approved by the local Research Ethics Committee, and all participants gave their written informed consent according to the Declaration of Helsinki.

All participants underwent a detailed clinical examination, and demographic data were collected by a questionnaire. Clinical parameters including duration and type (limited/diffuse) of the disease, the presence of severe complications [pulmonary arterial hypertension (PAH), interstitial lung disease (ILD), myocardial fibrosis], current medical treatment, and CV comorbidities (arterial hypertension, dyslipidemia, obesity) were also recorded. Arterial hypertension was defined as office blood pressure (BP) ≥ 140/90 mmHg, or history of antihypertensive drug intake [13]. Laboratory parameters such as routine biochemistry and hematology, lipid and bone profile tests, erythrocyte sedimentation rate (ESR), C-reactive protein (CRP), antinuclear (ANA), anti-topoisomerase (anti-Scl-70), and anticentromere (ACA) autoantibodies were also measured.

### Nailfold video-capillaroscopy

NVC was performed for the assessment of microcirculation damage by one operator (ET) according to standard protocols [14]. The nailfolds of all fingers, excluding thumbs, were examined bilaterally using a video-capillaroscope with a 200× contact lens. Patients remained for 20 min in a room with a temperature between 20 and 25 °C before the nailfold examination. An area of 1 mm^2^ per finger was studied. Pictures were collected, registered, and analyzed with image analysis software (Optipix-Optilia Instruments AB, Sweden). In every image, the following capillary abnormalities were evaluated: ramified and bushy capillaries, enlarged and giant capillaries, areas of capillary loss, microhemorrhages, and the presence of edema.

According to NVC results, patients were categorized in one of the following qualitative patterns: i.e., early, active, and late NVC pattern [15]. The early NVC pattern included well-preserved capillary architecture with a few detected capillaroscopic abnormalities, i.e., enlarged/giant capillaries and capillary microhemorrhages. The active NVC pattern included moderate capillary loss and numerous capillaroscopic changes, i.e., capillary enlargement, ramification, microhemorrhages, and subcutaneous edema. Finally, the late NVC pattern included advanced microangiopathy, i.e., extensive areas of capillary loss (avascular areas), complete disorganization of capillary distribution, and rare, abnormal, usually ramified, or bushy capillaries. Quantitative assessment of the capillaroscopic findings included capillaries per mm^2^. Furthermore, the mean capillaroscopic skin ulcer risk index (CSURI), according to the formula *D* × *M*:*N*^2^ (*D* maximum diameter of giant capillaries, *M* number of giant capillaries, and *N* total number of capillaries in the distal row), was calculated for each participant. CSURI was recently proposed as a novel prognostic capillaroscopic marker, able to predict the development of digital ulcers in SSc, with higher values being indicative of more extensive vascular damage [16]. The calculation of CSURI was automatically performed by the software image analysis.

### Surrogate markers of arteriosclerosis

The following markers of vascular function were measured for each patient. Bilateral B-mode ultrasonography was performed by an operator (NK) blinded to the NVC findings and the estimated CV risk of the examined patient, for the measurement of cIMT according to standardized protocols [17]. A General Electric Vivid-7 ultrasound device equipped with a linear array transducer was used, and at least three measurements were performed in the far proximal wall across a 5-mm segment of both right and left common carotid arteries to derive the mean cIMT. Augmentation index (AIx) and PWV were measured for the assessment of arterial stiffness. Carotid-femoral PWV was determined with the SphygmoCor device (AtCor Medical, Sydney, Australia) [18] and was estimated by the transit time between carotid and femoral pressure with respect to the electrocardiogram. Higher values of PWV are indicative of increased arterial stiffness. Using the integrated software, the central systolic and diastolic blood pressure (CSBP and CDBP) as well as the heart rate was evaluated. AIx was defined as the ratio of the second to the first peaks from the recorded pulse waveform, expressed as a percentage of the pulse pressure, providing a measurement of the reflected pulse wave and the arterial stiffness [19]. As there is a linear correlation between heart rate and AIx and in order to achieve comparable measurements, the adjusted AIx to a heart rate of 75 beats per minute (AIx-75) was recorded.

### Statistical analysis

Statistical analysis was performed using SPSS for Windows (version 22.0 IBM Corp: Armonk, NY, USA). Descriptive statistical tests were used for the presentation of the cohort’s main characteristics. The Shapiro-Wilk normality test was performed for the evaluation of the distribution of the quantitative variables. Those normally distributed were expressed as mean ± standard deviation (SD) while median values and range were used to describe variables not normally distributed. Categorical variables were expressed as frequencies and percentages. Correlation analysis between quantitative variables was performed with Pearson’s coefficient and Spearman’s rank order, depending on the normality of the distribution. To compare numerical means between two or more independent groups (for example between the three scleroderma patterns), the Student’s *t* test or one-way ANOVA analysis with Bonferroni correction, or the Mann-Whitney and Kruskal-Wallis tests were performed, according to normality. The *χ*^2^ test or Fischer’s exact test, as appropriate, was used for the analysis of categorical data. Adjustment for factors assumed to potentially affect the observed associations, including specific medication (i.e., endothelin receptor antagonists, phosphodiesterase inhibitors, and calcium channel blockers (CCBs)), disease duration, and arterial hypertension, was conducted via partial correlations. Due to the small sample size, control for each confounding factor (covariate) was performed separately. Statistical significance was set at a two-tailed *p* value of 0.05.

## Results

### Patient characteristics

In total, 37 SSc patients (36 female) were included in the present study. The mean age of our cohort was 55.2 ± 12.9 years and the median disease duration was 9 (0.5–42) years. Demographic data and disease characteristics are summarized in Table 1. Thirteen patients were classified through clinical assessment as diffuse SSc. ILD was present in 14 (37.8%) patients, while 8 (21.6%) had PAH. Regarding laboratory tests, anti-Scl-70 were positive in 13 (35.1%) and ACA in 16 (43.2%) patients. The mean ESR was 23 ± 18 mm/h. By the time of their first evaluation, 10 patients were taking endothelin receptor antagonists (bosentan or ambrisentan) and 2 phosphodiesterase inhibitors for either PAH or digital ulcers. More than half of the participants (51.4%) were on CCBs as treatment for arterial hypertension (9 patients), for Raynaud phenomenon (8 patients), or for both (2 patients), while 11 patients were found to have elevated pressure values as defined above.

**Table 1:** Demographic, clinical, and biochemical characteristics of the patients

Parameter	Values
*N*	37
Age (years)	55.2 ± 12.9
Female (*n*, %)	36 (97.3%)
Disease duration (years)	9 (0.5–42)
Skin ulcers (*n*, %)	12 (32.4%)
ESR (mm/h)	22.6 ± 18
Total cholesterol (mg/dL)	195 ± 41
Creatinine (mg/dL)	0.79 ± 0.25
ANA + (*n*, %)	33 (89.1%)
Anti-Scl-70 + (*n*, %)	13 (35.1%)
ACA + (*n*, %)	16 (43.2)
Type of the disease (*n*, %)	
Diffuse	13 (35.2%)
Limited	24 (64.8%)
PAH	8 (21.6%)
Interstitial lung fibrosis (*n*, %)	14 (37.8%)
NVC patterns (*n*, %)	
Early	11 (29.7%)
Active	10 (27.0%)
Late	16 (43.2%)
CSURI	4.2 (0.63–34.6)
cIMT (mm)	
Right	0.74 ± 0.14
Left	0.68 (0.52–1.16)
PWV (m/s)	7.2 (4.8–18.5)
AIx (%)	30.2 ± 11.6
CSBP (mmHg)	121 ± 20
SBP (mmHg)	131 ± 21
DBP (mmHg)	76 ± 10
Calcium channel blockers (*n*, %)	19 (51.4)
Vasodilators	
ERAs (*n*, %)	10 (27%)
PDEi (*n*, %)	2 (5.4%)

*ESR* erythrocyte sedimentation rate, *PAH* pulmonary arterial hypertension, *CSURI* capillaroscopic skin ulcer risk index, *cIMT* carotid intima-media thickness, *PWV* pulse wave velocity, *AIx* augmentation index, *CSBP* central systolic blood pressure, *SBP* systolic blood pressure, *DBP* diastolic blood pressure, *ERAs* endothelin receptor antagonists, *PDEi* phosphodiesterase inhibitors

Normally distributed continuous variables are expressed as mean ± standard deviation, whereas those not normally distributed are expressed as median (range)

Regarding NVC measurements, all patients presented pathological alterations of the nailfold capillary bed. More specifically, 11 (29.7%) patients had an early, 10 (27%) an active, and 16 (43.2%) a late scleroderma pattern.

### Clinical characteristics of NVC patterns

Increasing rates of internal organ involvement were observed among worsening NVC patterns. A diagnosis of PAH was present in 30%, 0%, and 35.7% of patients with an early, an active, and a late NVC pattern, respectively. Among patients with the late pattern, 53.8% had ILD compared to 44.4% in the active and 30% in the early pattern. Similarly, esophageal involvement was more common in the late NVC pattern (28.6%) compared to the early (10%) and the active (11%) patterns. Nevertheless, these discrepancies regarding visceral organ involvement did not reach statistical significance in between-group statistical comparisons. The prevalence of arterial hypertension, diabetes mellitus, and smoking did not significantly differ between the three groups. No differences regarding disease duration were either found. In contrast, a significant association was found between anti-Scl-70 antibodies and the late NVC pattern. Positive anti-Scl-70 antibodies were found in 78.6% of patients with a late pattern, compared to 14.3% and 7.1% of patients with an active and an early scleroderma pattern, respectively (*χ*^2^ = 11.016, *p* = 0.004). Regarding disease duration, no differences were found between the three groups.

### NVC patterns and vascular markers

**Fig. 1:**
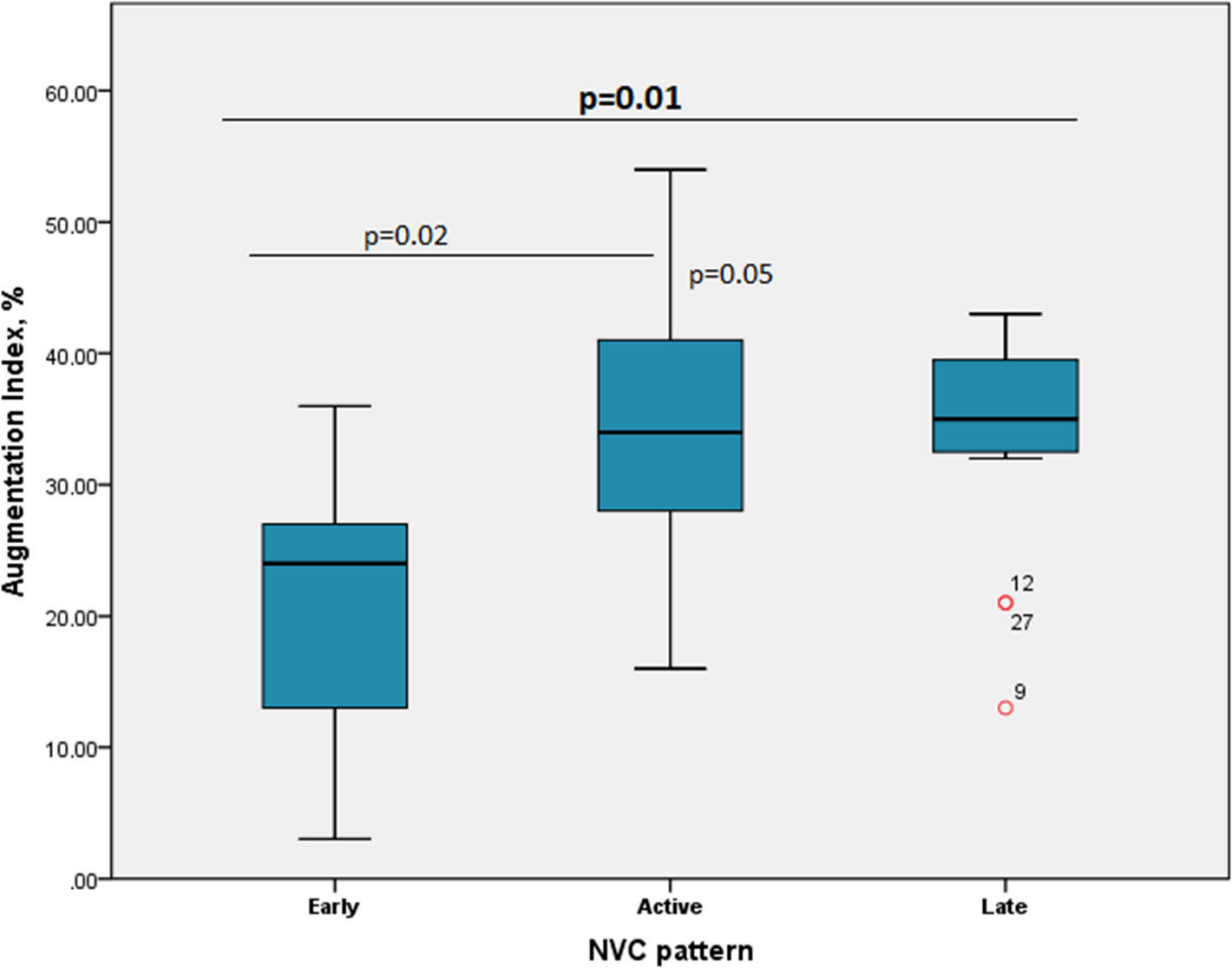
Augmentation index in patients with different nailfold video-capillaroscopy (NVC) patterns. Data are given as 5th, 10th, 50th (median), 90th, and 95th percentiles

Comparisons among markers indicative of macrovascular disease in the different NVC patterns are presented in Table 2. A significant correlation was observed between higher AIx-75 values and capillaroscopic patterns representing more severe microvascular damage (*p* = 0.01). Particularly, in between-group comparisons, both patients with an active or late NVC pattern had significantly higher AIx-75 compared with those with an early NVC pattern [early, 20.5 ± 11.4, active, 34.1 ± 11.5 (*p* = 0.02 vs early), late, 33.4 ± 8.8% (*p* = 0.05 vs early) (Fig. 1). Controlling for both disease duration or current medication for PAH or digital ulcers did not affect the significance of the observed associations. Age was also not found to affect the association between AIx-75 and NVC patterns. cIMT and PWV values as well as CSBP and CDBP did not differ between the three NVC patterns. No association between markers of inflammation (ESR and CRP) and surrogate markers of macrovascular disease was found.

**Table 2:** Surrogate markers of macrovascular disease in the NVC pattern categories

Parameter	Scleroderma pattern						
	Early	Active	Late	*p* (early vs active)	*p* (early vs late)	*p* (active vs late)	*p*
CIMT right (mm)	0.76	0.69	0.76				*p* = 0.466
CIMT left (mm)	0.67	0.69	0.80				*p* = 0.165
CSBP (mmHg)	119	122	122				*p* = 0.937
CDBP (mmHg)	75	82	75				*p* = 0.337
AIx (%)	20.5	34.1	33.4	*p* = 0.02	*p* = 0.05	*p* = 0.87	*p* = 0.001
PWV (m/s)	7.3	6.9	8.0				*p* = 0.566

*CIMT* carotid intima-media thickness, *CSBP* central systolic blood pressure, *CDBP* central diastolic blood pressure, *AIx* augmentation index, *PWV* pulse wave velocity

### Quantitative rating of NVC findings and vascular markers

**Fig. 2:**
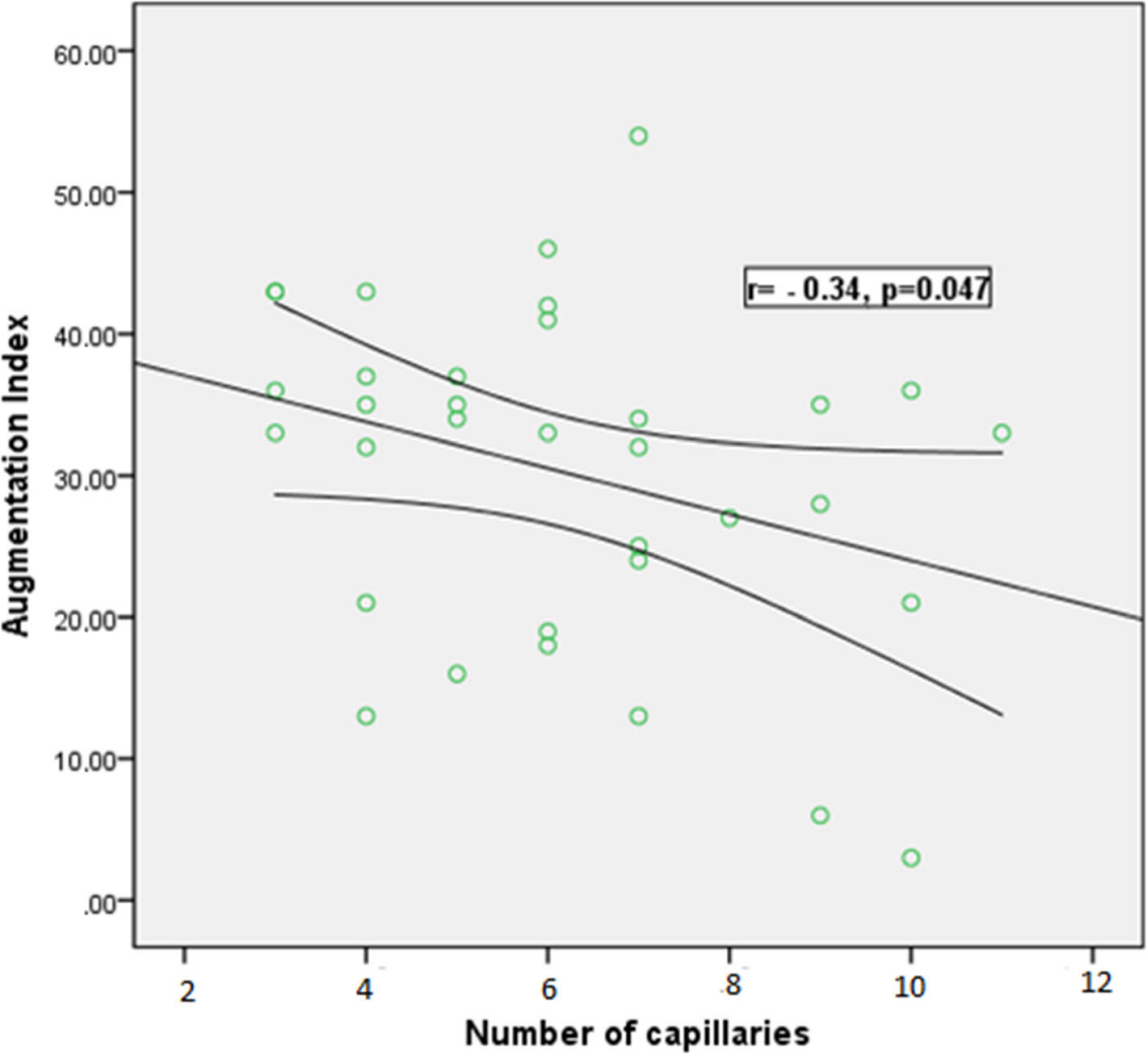
Inverse correlation between augmentation index (%) and the number of capillaries, as assessed by nailfold video-capillaroscopy

**Fig. 3:**
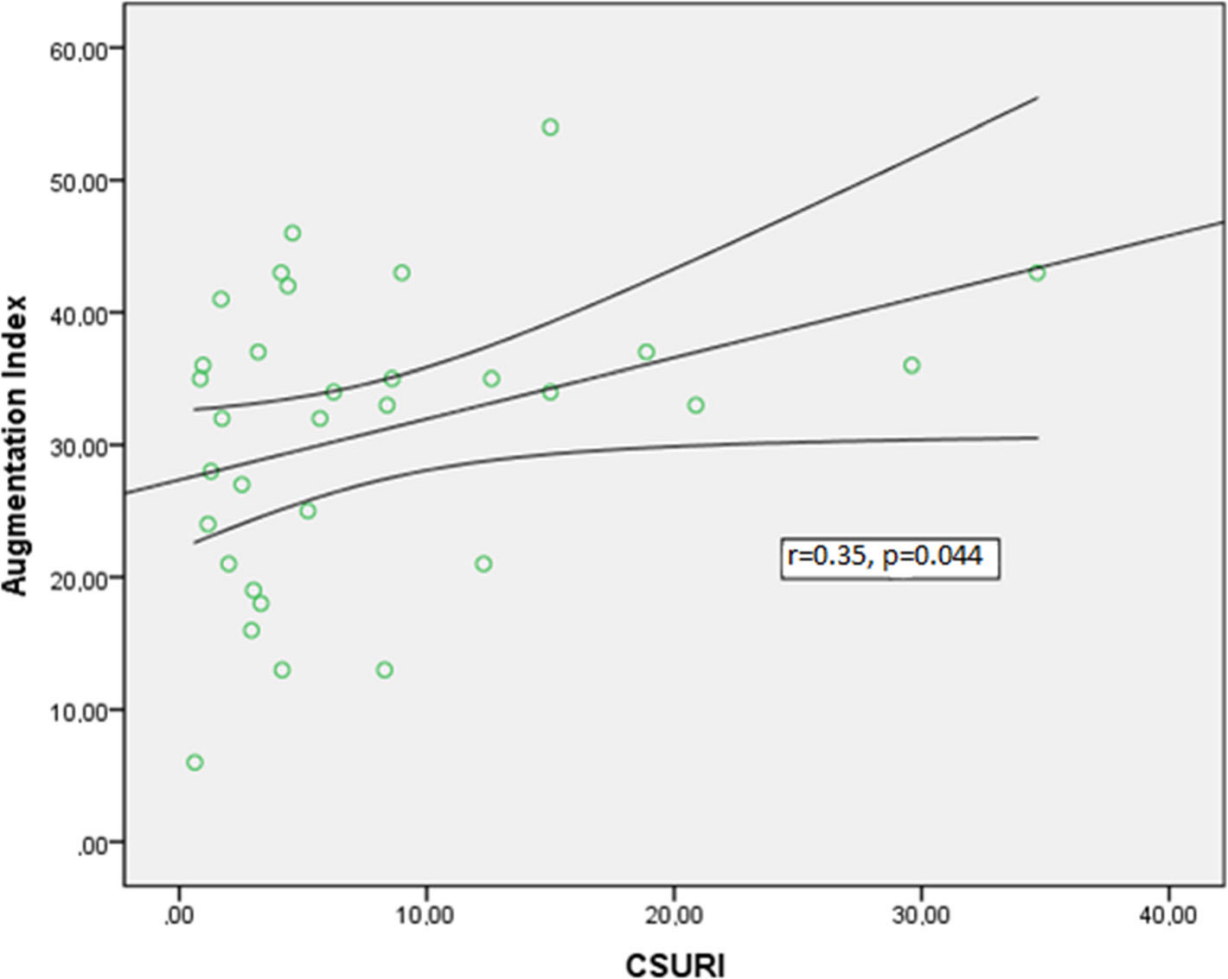
Positive correlation between augmentation index (%) and CSURI index, as assessed by nailfold video-capillaroscopy

Correlation analysis between the mean number of capillaries per mm^2^ (expressing capillary density), the CSURI index, and parameters reflective of microcirculation is presented in Table 3. A moderate negative correlation was observed between the number of capillaries per mm^2^ and ΑΙx-75 (*r* = − 0.34, *p* = 0.047); higher AIx-75 values were associated with fewer capillaries per mm^2^ and, hence, lower capillary density (Fig. 2). In contrast, the prognostic capillary index CSURI presented a moderate positive correlation with AIx-75 (*r* = 0.35, *p* = 0.044) (Fig. 3). No other significant associations were found between surrogate markers of atherosclerosis and capillaroscopy changes (Table 3).

**Table 3:** Quantitative rating of capillaroscopic findings and surrogate markers of macrovascular disease

Parameter	Correlation (*r*)—significance (*p*)	
	Number of capillaries per mm^2^	CSURI
CIMT right	*r* = 0.159, *p* = 0.360	*r* = − 0.107, *p* = 0.561
CIMT left	*r* = 0.03, *p* = 0.985	*r* = − 0.073, *p* = 0.692
CSBP	*r* = 0.139, *p* = 0.425	*r* = 0.032, *p* = 0.861
CDBP	*r* = − 0.034, *p* = 0.845	*r* = 0.272, *p* = 0.131
AIx	*r* = − 0.343, *p* = 0.047	*r* = 0.358, *p* = 0.044
PWV	*r* = 0.130, *p* = 0.463	*r* = − 0.252, *p* = 0.164

*CIMT* carotid intima-media thickness, *CSBP* central systolic blood pressure, *CDBP* central diastolic blood pressure, *AIx* augmentation index, *PWV* pulse wave velocity

Adjustment for the treatment with endothelin receptor antagonists, phosphodiesterase inhibitors, and CCBs or for disease duration did not affect these correlations. Although age was found to significantly associate with cIMT (*r* = 0.533, *p* = 0.001), PWV (*r* = 373, *p* = 0.025), and AIx-75 (*r* = 0.530, *p* = 0.001), adjustment for age affected neither the correlation between AIx and capillary density nor the correlation between AIx and CSURI. Finally, there was no significant difference in AIx values between patients with and those without arterial hypertension and, hence, no alterations in the aforementioned associations were observed when controlling for arterial hypertension.

## Discussion

The main findings of our study are the significant associations between both capillary density and CSURI index with arterial stiffness indices, suggesting a possible association between micro- and macrovascular injury in SSc. AIx-75 was significantly higher in patients with the active and late NVC pattern compared to those with the early NVC pattern. Our results indicate a progressive increase of arterial stiffness and, thereby, macrovascular disease, among higher grades of SSc microangiopathy.

Functional and structural alterations in the microvasculature, resulting progressively in an excessive capillary loss, represent the main pathogenetic process in SSc [20]. Previous research has demonstrated a respective involvement of micro- and macrovascular dysfunction in SSc. Macrovascular function, assessed by brachial artery FMD, was found to be impaired in patients with the early NVC pattern compared with controls [11]. Lower FMD values were recorded in patients with the late compared with the early and active NVC patterns (*p* = 0.003 and *p* = 0.001, respectively) [11]. Such findings highlight the presence of an association between macrovasculopathy and the progression rate of damage in the microvascular bed. With regard to structural damage, the perfusion pattern of the proper palmar digital arteries is disturbed in SSc patients with demonstrated microvasculopathy, showing a gradual reduction among worsening NVC patterns, since structural changes detected by Doppler ultrasonography were present in the later stages of SSc microangiopathy [21]. Similarly, a larger study in 115 Spanish SSc patients revealed that morphological markers of subclinical atherosclerosis (assessed by carotid ultrasound) were more prevalent across patients with avascular areas in NVC [10]. Taking all these data together, functional markers may be more reliable than structural changes for the assessment of macrovasculopathy in the early stages of SSc.

Arterial stiffening has been reported in SSc patients compared with controls [22], but a study assessing arterial stiffness by PWV did not report any significant difference between PWV values and different NVC patterns in 39 SSc patients [12]. In contrast, the prospective multi-center ERAMS study suggested that abnormal macrovascular function may reflect vascular damage and worse prognosis in SSc patients by establishing a relationship between AIx-75 and the extend of lung disease [23].

Our findings indicate a progressive increase of arterial stiffness, in correlation with worsening phases of SSc microangiopathy, particularly in the presence of the late (worst) NVC pattern, supporting the hypothesis that impairment of vascular elasticity and peripheral artery resistance could be related with microvascular damage in SSc as it has been reported in hypertensive patients [24]. Whether the association between capillaroscopic changes and arterial stiffening demonstrated in our study suggests that NVC could contribute—among others—to CV risk stratification in SSc population remains to be determined in larger studies.

Furthermore, the capillaroscopic index CSURI, which was designed to predict the formation of new digital ulcerations, was found to positively correlate with AIx-75. In line with our study, Aissou et al. reported that the presence of digital ulcers is associated with increased AIx-75 whereas there was no relation with PWV [25]. While the aortic PWV represents a direct measure of the elastic properties of the aorta, aortic augmentation derives from the contribution of peripheral wave reflection to the central arterial pressure waveform and depends on the geometry and vasomotor tone of smaller arteries [26]. Hence, the observed correlation between CSURI and AIx-75 may suggest that mainly small and medium artery stiffening, rather than large artery stiffening, is associated to peripheral microvascular disease in SSc [27].

Our cohort was quite heterogeneous regarding the duration of SSc (ranging from 0.5 to 42 years). Similarly to what is known for the general population, age and blood pressure seem to be the main determinants of large artery stiffening in SSc, measured by PWV [28], while the effect of disease duration on PWV remains controversial [29]. The observed association between AIx-75 and capillaroscopic measurements also remained unaffected after adjustment for disease duration, suggesting that additional pathogenetic mechanisms, such as disease activity and severity rather than time alone, contribute to the concomitant evolution and natural course of micro- and macrovascular involvement.

Vasoactive medications reported to interfere with the progression of nailfold microvasculopathy [30] were also not found to have a significant impact on our findings. It should be noted that statins and antihypertensive drugs may affect arterial stiffness [31, 32]. However, none of our patients was on statins or antihypertensive drugs other than CCBs, and treatment with CCBs did not appear to influence the results.

There are several potential limitations to be recognized in our study. This is a single-center study with a rather small patient sample, which may limit the power of our statistical analysis. We investigated specific changes of the capillary bed, and we chose both qualitative and quantitative methods to document our findings. NVC is an operator-dependent method, and all examinations were performed by one operator who was—however—blinded to other vascular parameters. Finally, we did not include measures of endothelial dysfunction, such as FMD, which could provide a more sensitive detection of subclinical atherosclerosis. On the other hand, we performed adequate morphological measurements of the vasculature in a real-life population representative of the SSc patients attending a routine rheumatology outpatient clinic.

## Conclusions

In conclusion, we found significant correlations between arterial stiffness and progressive microvascular damage (assessed by NVC) suggesting that micro- and macrocirculation might represent different morphological reproductions of disease progression in SSc patients, sharing common pathogenetic mechanisms. These preliminary data could constitute the basis for further research, investigating the relationship of vascular injury in different vascular beds in SSc contributing to the global evaluation of endothelial damage in this population.

## Data Availability

The data of the study, in the form of datasets, could be provided anytime after email communication with the authors (SS, EP, or TD).

## Abbreviations

ACA: Anticentromere (autoantibodies)
ACR: American College of Rheumatology
AIx: Augmentation index
ANA: Antinuclear (autoantibodies)
cIMT: Carotid intima-media thickness
CCBs: Calcium channel blockers
CDBP: Central diastolic blood pressure
CSBP: Central systolic blood pressure
CSURI: Capillaroscopic skin ulcer risk index
CRP: C-reactive protein
CVD: Cardiovascular disease
ESR: Erythrocyte sedimentation rate
EULAR: European League Against Rheumatism
FMD: Flow-mediated dilatation
ILD: Interstitial lung disease
NVC: Nailfold video-capillaroscopy
PAH: Pulmonary arterial hypertension
PWV: Pulse wave velocity
SSc: Systemic sclerosis
Scl-70: Anti-topoisomerase (autoantibodies)
